# Attachment and the Development of Moral Emotions in Children and Adolescents: A Systematic Review

**DOI:** 10.3390/children8100915

**Published:** 2021-10-14

**Authors:** Mariana Costa Martins, Ana Filipa Santos, Marília Fernandes, Manuela Veríssimo

**Affiliations:** William James Center for Research, ISPA-Instituto Universitário, 1100-304 Lisboa, Portugal; mariana.g.c.martins@hotmail.com (M.C.M.); ana.f.santos2@hotmail.com (A.F.S.); mfernandes@ispa.pt (M.F.)

**Keywords:** attachment, social-emotional development, moral emotions, empathy, guilt

## Abstract

In recent years, the development of social and moral emotions (often associated to pro-social behaviors) has become the subject of increased research interest. However, the relation between these emotions and attachment is less studied. The present systematic literature review (PROSPERO: CRD42021247210) was designed to synthesize current empirical contributions that explore the link between attachment and the development of moral emotions (e.g., empathy, sympathy, altruism, and guilt) during childhood and adolescence. Article exclusion criteria included: studies with participants not living in natural contexts (e.g., institutionalized); studies on mental illness; qualitative research; research that does not reliably evaluate attachment or moral emotions; research on intervention programs; and non-peer-reviewed articles. Only 10 studies were found eligible. Results highlight a present focus on empathy and guilt and gaps regarding sympathy and altruism. The mediator role and positive effect of emotion regulation was noted. Significant positive correlations between attachment security and guilt, shame and forgiveness were emphasized. Limitations of the eligible studies included: representativeness of the participants; causality of the results; and the validity and significance of the instruments (e.g., lack of results reported by various parties involved). The present review aims to contribute to the understanding of an empathic, healthy development, in contrast to the alienation and bullying affecting the youth’s emotional, relational and academic lives.

## 1. Introduction

Secure base relations are fundamental for socioemotional development [1,2,3,4]. Children who experience consistent and sensitive caregiving develop internal secure working models of the self and of relationships that will guide different aspects of social development, such as emotional regulation or social competence [5,6,7]. For example, the internal working model will shape the child’s perception of emotional expression adequacy, that is, the expression effectivity in eliciting sensitive responses from others to child’s needs [3]. These internal working models can also manifest in differences in the individuals’ emotional and behavioral responses to others’ distress [8], guiding prosocial development and empathic responses. Previous research showed a relation between moral emotions, specifically greater empathy, sympathy, guilt and more pro-social behavior. Moral emotions can be considered as one of the foundations for pro-social behaviors and necessary for their emergence [9,10]. Similar results were found in adolescence, with attachment security being associated with greater empathy and greater vagal tone (which, in turn, is associated with greater emotion regulation skills involved in social behaviors [11]), the latter during maternal interactions [12].

Despite the vast empirical support for the association between attachment and social development [5,13], less is known about the relations between attachment and the development of moral emotions.

Most studies focus on other aspects of family functioning and the child–parent relationship (e.g., parental support, [14]; positive parenting, [15], parental warmth, [16]), even though the significant effects when attachment is studied together with these dimensions [13].

### 1.1. Empathy, Sympathy and Pro-Social Behavior

Moral emotions can guide behavior or be an anticipation as a result of an evaluation of behavioral alternatives [13,17,18], reflecting previous emotional experiences as well as expectations. These emotions have an important adaptive function and might prevent moral or conduct transgressions by making the child aware of possible negative consequences for himself and the other [19]. For example, the child’s ability to feel sympathy in anticipation of or in response to another’s suffering might inhibit the child’s engagement in aggressive actions that would harm others [20]. 

Sympathy is defined as a feeling of concern for others, which often, but not always, results from a shared emotional state or experience of distress. It derives from the cognitive awareness of the state or condition of the other [21,22], directing the person’s attention to the consequences of his or her actions—to the given rights or well-being of the other—and is conceptually related to costlier altruistic forms of pro-social behavior [23]. Despite the conceptualization of sympathy as a protective factor, researchers often use empathy-related response measurements that do not differentiate sympathy from empathy and personal distress [21,24]. Sympathy can develop from empathy; however, it is important that there is a distinction between the two concepts [21].

Empathy is a fundamental component of social competence and a known precursor to moral reasoning. It is conceptualized as the ability to accurately perceive and respond to another person’s feelings, emotions and affective states [25,26]. Therefore, it is plausible to presume that empathy plays an essential and critical role in close relationships by promoting mutual understanding and sensitivity [27,28]. Positive social emotions, such as empathy, concern for others, compassion or sympathy, have behavioral and social implications by promoting helping or sharing behaviors and inhibiting aggressive actions, all of which can improve the quality of relationships [23,29,30,31]. 

### 1.2. Guilt and Reparative Behavior in Relationships

The self-conscious emotion of guilt refers to a feeling of tension, remorse and regret over an inappropriate behavior in presence of other people. Both guilt and shame have been defined as prototypical moral emotions that guide compensatory behaviors in cases of condemnable social actions committed by oneself [32]. Similar to empathy and sympathy, guilt and shame are often used interchangeably in the literature, and it is crucial to distinguish between both concepts [33]. Guilt is the result of a negative evaluation of one specific behavior (“I did that wrong”); shame is also related to a negative evaluation but referring to the global self (“I did/was wrong”). This implies that in guilt there is a feeling of regret and remorse and a wish that oneself behaved differently to prevent or undo the harm done [34]. These differences are also manifested in the functionality of both emotions, since guilt motivates reparative behavior (i.e., making apologies and engaging in attempts to fix the situation), while shame motivates a more defensive, avoidant and submissive behavior [35].

Guilt is generally described as having an adaptive value, although the dysregulation of this emotion has been associated with several psychopathological symptoms. High levels of self-conscious emotion might be maladaptive, for example, when guilt is experienced in an obsessive, ruminative way or fused with feelings of shame [36]. On the other hand, it has been argued that guilt can also promote socially reparative behaviors after transgressions, playing an important role in the development of empathy and conscience [14,37]. This last formulation of guilt will be considered in this work, since there is increasing evidence showing that socially problematic behaviors (such as aggressive ones) are associated with lower levels of guilt e.g., [38,39].

Moral emotions might be rooted in early affective experiences with attachment figures e.g., [40,41]. Studies on empathy and prosocial development have progressed largely in parallel with the flourishing research on attachment, and obvious points of intersection have been articulated [25,42,43]. 

The present systematic literature review aims to explore and synthesize the relation between attachment and moral emotions (e.g., empathy, sympathy, altruism, and guilt) during childhood and adolescence, while answering the following question: How does attachment relates to empathic, social and moral emotions in preschoolers, middle school children and adolescents? Is quality of attachment related to empathy (and other moral emotions such as sympathy, altruism and guilt)? Is that effect indirect or mediated through other important variables (e.g., emotion regulation [6])? This is a field of recent interest in the literature, but it is essential for understanding how to support healthier, more empathic, sympathetic and pro-social development of children’s emotional lives, in contrast to the increasing alienation, bullying (also reported in the literature [44]) that is affecting children’s relational, emotional and academic lives.

## 2. Materials and Methods

This review follows the general guidelines presented in Preferred Reporting for Systematic Reviews (PRISMA, [45]) to analyze the relation between attachment and moral emotions (specifically: empathy, sympathy, guilt and altruism). 

**Figure 1:**
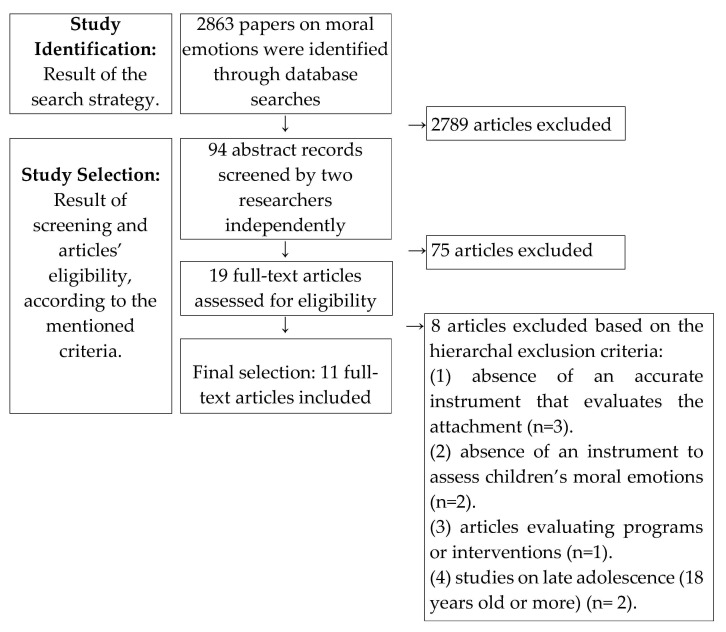
Flowchart of the study identification and selection process (according to the PRISMA [45] guidelines).

Prior to data extraction, this review protocol was registered on the International Prospective Register of Systematic Reviews (PROSPERO; registration number: CRD42021247210). The identification, screening and eligibility verifying process is synthesized in Figure 1, and each of these steps will be detailed next. 

### 2.1. Search Strategy and Article Eligibility Criteria 

First, articles’ titles were screened, duplicates were removed and relevant studies were selected and exported. For this purpose, a set of inclusion and exclusion criteria were established for article selection (Table 1). For abstract screening, the following inclusion criteria were established a priori: (1) empirical articles with available abstract published in peer-review journals; (2) articles published in Portuguese, English, French or Spanish (languages mastered by the authors); and (3) articles examining the relationship between attachment and moral emotions (i.e., sympathy, empathy, guilt or altruism). For the remaining articles, abstracts were screened by the main researcher and other two independent reviewers to assess whether the paper met the eligibility criteria; those that did not were excluded. If the information required to determine eligibility was not available from the title and abstract, the full text was screened. 

Exclusion was established a posteriori (Table 1): (1) children or adolescents not living in natural contexts (e.g., institutionalized children); (2) studies of moral emotions in the context of mental illness or addictive substance usage; (3) intervention programs; (4) articles aiming to develop, adapt or validate measures of moral emotions; (5) studies with a qualitative design; and (6) non-peer-reviewed articles (e.g., book chapters, conference papers or posters and studies measuring attachment that did not follow Bowlby’s [46] or Ainsworth and colleagues’ [1] conceptualization of the construct). In terms of the selection process, articles that related attachment to pro-sociality were included, and a chance was given to include articles that addressed its opposite: bullying.

Three reviewers were involved in data extraction. Disagreements and discrepancies were discussed until consensus was reached. If consensus was not achieved, another(s) reviewers(s) were consulted.

Studies that looked at empathy and moral emotions in particular cases of psychopathology such as psychopathy and autism were excluded. Other common issues related to empathy such as morality e.g., [47], therapeutic interventions e.g., [48], politics and social issues such as racism e.g., [49] and care workers’ well-fare and empathy e.g., [50] were also excluded.

This systematic data search was performed in EBSCO’s databases (e.g., PsycINFO, Psychology and Behavioral Sciences Collection) using the following search terms (combined with Boolean terms): attachment AND (moral emotions OR empathy OR sympathy OR guilt OR altruism). The combination of these terms was searched in the title, abstract and keywords. The search was applied to the last 30 years (until 15 July 2021) and resulted in 2856 records. To this number, 7 other articles of interest were identified through cross-referencing, making a total of 2863 articles screened (Figure 1). 

### 2.2. Study Selection Procedures

The initial 2863 articles were screened according to the established inclusion criteria by the first author and 2789 articles were excluded at this stage. The remaining 94 articles were screened by the first and second author to assess eligibility for inclusion according to the criteria listed above, and 19 full-texts were further assessed independently by the first 2 authors for inclusion. Discrepancies were resolved by consensus. After full-text review by the first 2 authors, 10 articles (see Appendix A) met all the inclusion criteria (Figure 1).

### 2.3. Data Extraction Procedures

A categorization system was developed to collate and summarize the results. The categorization system was developed to identify: (1) general characteristics of the studies, for example, country of origin, theoretical background (Table 2); (2) general characteristics of the participants, for example, ethnicity, socioeconomic background, age range (Table 2); and (3) assessments of moral emotions, e.g., sympathy, guilt (Table 2). The classification of the retrieved articles was performed by the first two authors. Discrepancies were discussed until consensus was reached.

## 3. Results

### 3.1. General Description of the Studies: Theoretical and Empirical Perspectives

All selected articles used as theoretical background developmental psychology theories, such as attachment theory and social-emotional development, and referenced the main founders of these theories. Only studies with original data were selected (Table 2). Four studies used a longitudinal design (40%). Most studies used child-reported measures while a minority used observational measures. This was true for both the moral emotion measures (child-reported: 63.64%) and the attachment measures (child-reported: 63.64%), (Table 2).

### 3.2. General Characteristics of the Sample and Assessments

Most of the studies were conducted in Anglo-Saxon countries (United States of America—63.64%). Studies in childhood and adolescence proved to be evenly distributed (45.45% and 54.55%, respectively). Most of the samples revealed a majority of medium-high economic status participants (50%), (Table 2). 

Within this theoretical context, the most studied moral emotion was empathy (69.23%), followed by guilt (15.38%), forgiveness (7.69%) and shame (7.69%) (Table 2). No studies were found regarding the potential link suggested by the literature between attachment and sympathy or altruism.

Further individual assessment on the selected articles’ participants and instruments are presented in Table 3:

### 3.3. Findings on the Different Influences between Attachment and Moral Emotions

The results regarding infancy showed that from 16 to 22 months, empathic concern for mother’s distress increased, whereas empathy for strangers decreased. A more fearful temperament and less attachment security predicted less empathic concern for stranger’s distress [13].

Among the 10 articles selected, Murphy and colleagues’ [59] study presented positive correlations between attachment and emotion regulation (0.30, *p* < 0.01) and also empathy (0.17, *p* <0.05), guilt (0.26, *p* < 0.01) and forgiveness (0.33 *p* < 0.01). In Muris and colleagues’ [10] study there was significant differences in the children’s shame (F = 7.92, *p* = <0.001) across different attachment styles but not in guilt (F = 2.11, *p* = 0.12); the avoidant style had higher levels of shame.

It was also possible to identify five pathways of analysis and significant mediation models [6,12,52,64,67]. These models, in summary, revealed that the quality of attachment had a positive and significant association with empathy, and that empathy was a significant mediator of the indirect effect between maternal attachment and pro-social behavior [52]. Emotional regulation was a significant mediator of the effect of attachment on empathy levels, revealing that more secure children were rated higher in emotional regulation and, consequently, higher in empathy [6]. Children with below-average emotion regulation strategies showed a larger effect of attachment security on empathy, while children with above-average emotional regulation strategies and attachment security were the most empathic [64].

Secure attachment at 14 years of age predicted teens’ greater capacity to provide empathic support during observed interactions with friends across ages 16 to 18. Less secure teens were slower to develop these skills. Furthermore, teens’ attachment security predicted the degree to which friends called for their support, which was associated with teens’ responsiveness to such calls [67]. In addition, during adolescence, the highest levels of empathic sensitivity were found among youths with low attachment anxiety and high emotion regulation, whereas the lowest levels of empathic sensitivity were found among participants with high attachment anxiety and high emotion regulation, suggesting that the ability for emotion regulation might only facilitate empathic sensitivity among teens with low attachment anxiety [12].

Secure girls perceived a higher acceptance rate in the relationship with their parents (mothers: *p* ≤ 0.001; fathers: *p* ≤ 0.001), showed higher empathy (mothers: *p* = 0.018; fathers: *p* = 0.016) and obtained higher scores in perspective taking (mothers: *p* ≤ 0.001; fathers: *p* = 0.006) and empathic concern (mothers: *p* = 0.024; fathers: *p* = 0.022), [14].

Parent attachment had no direct links with social behavior. Instead, the link between parent attachment and social behavior was indirect, mediated by aspects of emotional competence (not only empathy but emotional awareness and positive expressiveness) [55].

## 4. Discussion

The present systematic review found a number of studies relating attachment to pro-social behavior and on the relation between moral emotions and pro-social behavior (symbolized here by the high number of articles found by EBSCO: 2863). However, the number is considerably lower (more specifically, 10), when addressing attachment and moral emotions and when instruments that reliably assess these variables are taken into account.

Regarding the 10 eligible articles, some limitations were found. The participants were mostly Caucasian and of medium-high socioeconomic status [64,67]. The research designs were mostly correlational and cross-sectional and did not allow us to verify a causal relationship between variables [10,52]. The same problem was found associated with path analysis, this being a correlational statistical analysis in nature [52].

The absence of genetic considerations regarding the empathic ability of both the parents and the children/adolescents was stressed. The lack of consideration for the effect of empathy-promoting interventions on children and adolescents was also pointed out [68]. Paez and Rovella [14], Murphy and colleagues [67] and, finally, Muris and collaborators [10] identified self-reported measures as a major limitation, as well as the lack of comparison with significant adult reports. Additionally, no study addressed the possible differences between attachment to mothers and attachment to fathers for the development of moral emotions relying predominantly on maternal reports (e.g., [6]). It is argued overall in these articles that using all types of instruments, assessments and reports of both children and parents will promote more meaningful and significant results [64]. Finally, in contrast, studies such as that of Diamond and colleagues [12] report as a limitation the lack of objectivity of their selected observational measures.

It is worth noting the significant gap found in the literature: the absence of studies relating attachment to the development of social-moral emotions such as sympathy and altruism (as seen in Table 2). This gap is particularly interesting when taken into consideration the substantial number of published articles exploring the relations between prosocial behaviors and moral emotions (see also: [41,70,71]); this could be related with the above-mentioned difficulty in conceptually distinguishing the concepts of sympathy and empathy (i.e., the lack of instruments that accurately differentiate both variables).

Our results reveal a small number of longitudinal studies, which is surprising, considering the predominant developmental theoretical context of the selected studies, there was also a predominant use of self-reported measures (and not reported by parents, teachers or observational measures). No specific differences were found in the predominance of research on children or adolescents, both being equally rare. The present systematic review reveals a consistent link between moral emotions and attachment [10,59]; particularly notable are the different models relating empathy and attachment that have been found in our selected studies. In these models, the mediating role of empathy between attachment and the pro-sociality of the developing individual can be registered [52]. Crucial as well is the positive mediating role of emotional regulation in the relationship between empathy and attachment. An opposite effect was found for negative emotions from the child since attachment security did not correlate positively with this type of expressiveness on the part of the child [6]. These rare but highly informative and significant studies substantiate the need for continuing and deepening the study of these domains.

In the search process, preference was given to the following moral emotions: empathy, sympathy, guilt, and altruism. A limitation that can be pointed out to this approach is the absence of other emotions considered moral and social, such as regret, compassion, shame, and forgiveness. Although some of these emotions were found in the selected studies [10], the absence of these emotions in the Boolean terms may have prevented the selection of other articles. Furthermore, as a potential consequence, a small number of eligible articles was found (10), making interpretations of the results and associations between the different studies difficult. For this same reason, and since these are the main articles to delve into this research topic, it was not possible to compare and explore how the different measures relate to moral emotions, considering the number of studies with parent’s reports instruments and observational methodologies was significantly low (two). Will these differing measures relate to empathy or guilt in similar ways or reveal differing conclusions and point out to different research issues? These shortcomings provide an opportunity for future systematic reviews to also address others moral emotions (e.g., embarrassment).

Additionally, the elected studies devoted limited attention to other contributors to these emotions (e.g., sociodemographic variables such as gender, family configuration and exposure to others) and to children’s characteristics and abilities known to support the development of moral emotions, such as the ability to appropriately attribute mental states and emotions to themselves and others, or theory of mind (ToM), previously associated in the literature to empathy [72] and guilt [73]. How attachment and moral emotions relate and vary in their association with different variables is not covered in depth. There is also potential to address these gaps in future investigations.

To conclude, it is important to point out that the 10 articles and 11 studies proved to be coherent among themselves, despite differing methodologies, qualities and weights of results. Moreover, they supported what has been theoretically argued—the relationship between attachment and the socioemotional development of children and adolescents (more specifically, moral emotions). Securely attached children and teenagers demonstrate a greater ability to adequately express empathy and feelings such as guilt, forgiveness and shame.

Although only 10 studies addressed the association between attachment and moral emotions, we think it is an important and promising field of research. Understanding how these early relationships are related to the development of moral emotions might help to improve more tolerant and pro-social (and to decrease anti-social) behaviors in children, impacting their emotional, relational, and academic lives.

## Figures and Tables

**Table 1:** Summary of inclusion and exclusion criteria.

Inclusion Criteria	Exclusion Criteria
Empirical articles with available abstract published in peer-review journals; Articles published in Portuguese, English, French, Spanish or Italian (languages mastered by the authors); Articles examining the relationship between attachment and moral emotions and pro-sociality (e.g., sympathy and guilt, altruism); Articles with the following participants: Children and adolescents (0–19 years old).	Studies on children or adolescents not living in natural contexts (e.g., institutionalized children); Studies of moral emotions in the context of mental illness or addictive substance usage; Qualitive research; Research that does not accurately/directly evaluate parental attachment or moral emotions. Research on intervention programs; Non-peer-reviewed articles (e.g., book chapters, conference papers or posters).

**Table 2:** General characteristics of the selected articles.

Characteristics of the Studies	Total of Articles (*n*)	Percentage (%)	Articles ID ^a^
Theoretical background:			
Developmental psychology (socioemotional development)	10	100%	1–10.
Type of data:			
Original	10	100%	1–10.
Secondary	0	0%	-
Study design ^b^			
Longitudinal	4	40%	1, 2, 9, 10.
Cross-sectional	6	60%	3, 4, 5, 6, 7, 9.
Assessment of moral emotions			
Child/adolescent-reported	7	63.64%	1, 3, 4, 5, 6, 8, 9.
Parent-reported	2	18.18%	1, 7.
Observation	2	18.18%	2, 10.
Assessment of attachment			
Child/adolescent-reported	7	63.64%	1, 3, 4, 5, 6, 8, 9.
Parent-reported	2	18.18%	2, 7.
Observation	2	18.18%	2, 10.
Sample characteristics	*n* ^b^	%	ID ^a^
Country of origin			
Anglo-Saxon countries	7	63.64%	1, 2, 3, 4, 5, 7, 9.
European countries	3	27.27%	4, 8, 10.
South American countries	1	18.18%	6.
Age			
Child	5	45.45%	2, 4, 7, 8, 10.
Adolescent	6	54.55%	1, 3, 4, 5, 6, 9.
Social economic status			
High/Median	6	50%	1, 2, 4, 8, 9, 10.
Low	2	16.67%	1,4.
Not mentioned	4	33.33%	3, 5, 6, 7.
Characteristics of the assessment of moral emotions	*n* ^b^	%	ID ^a^
Empathy	9	69.23%	1, 2, 3, 5, 6, 7, 8, 9, 10.
Sympathy	0	0%	-
Guilt	2	15.38%	4, 5.
Altruism	0	0%	-
Others moral emotions:			
Forgiveness	1	7.69%	5.
Shame	1	7.69%	4.

^a^ Articles’ references are presented in the Appendix A. ^b^ According to inclusion criteria of the current review, only the quantitative results of studies with mixed methods were included.

**Table 3:** Summary of the selected articles’ samples dimensions, participants age and ethnicity and studies’ instruments.

ID. Authors (Date)	N	M Age (SD)	Ethnicity	Attachment Measure	Moral Emotion Measures
1. Diamond et al. (2012) [12]	103 (mother–adolscent dyads)	NA (14 years old)	82% Caucasian, 3% African American, 1% Asian, 7% Latino, 7% another or mixed ethnicity.	Adolescent Attachment Scale [51].	Empathy: During the re-viewing of their discussion task, participants rated positive and negative affect (5-point scale [11]).
2. Kim and Kochanska (2017): Family study [52]	101 families	NA	-	Strange Situation and Attachment Q-Set version 3.0 [53].	Empathy: Paradigm [54].
Laible (2007) [55].	117	19.6 years (1.41).	78% Caucasians	Inventory of Parent and Peer Attachment (IPPA) [56].	Empathy: Interpersonal Reactivity Index (IRI) [23].
4. Muris et al. (2014)—Study 1 [10]	688	10.39 years (1.00)	Majority: European descent (i.e., Dutch).	The Attachment Questionnaire for Children [57].	Guilt and shame: SCEMAS [58].
4. Muris et al. (2014)—Study 2 [10]	135	15.46 years (1.99)	Less than 10% was non-Caucasian	IPPA [56].	Guilt and shame: SCEMAS [58].
5. Murphy et al. (2015) [59]	148	15.68 years (1.16)	88.5% Caucasian, 5.4% Hispanic, 5.4% others	Shortened version of IPPA [56].	Empathy: Interpersonal Reactivity Index (IRI) [23].
6. Paez and Rovella (2019) [14]	518	15.22 years (1.69)	-	Kerns Security Scale (Argentinian adaptation [60])	Empathy: Interpersonal Reactivity Index (Argentinian adaptation) [61].
7. Panfile and Laible (2012) [6]	63 dyads	NA (3 months of age)	81% Caucasian.	Attachment Q-Set version 3.0 [53].	Empathy: My Child questionnaire [62] and Bryant’s Index of Empathy [63].
8. Ştefan and Avram (2018) [64]	212	56.34, months (11.52)	92.8% Caucasian, 0.5% Gypsy and 6.7% reported no ethnicity.	Attachment Security Completation Task [65].	Kid’s Empathic Development Scale (KEDS) [66].
9. Stern et al. (2021) [67]	184	14.27 (0.77) to 18.38 years (1.04)	58% Caucasian, 29% African American, 13% other	The Adolescent Attachment Interview [68] and Q-set [69].	Empathy: Observed supportive behavior task (SBT) [67].
10. van der Mark (2002) [13]	151	16–21 months	Mostly Caucasian	Strange situation	Empathy: observation coding system [19].

## Data Availability

Data sharing is not applicable to this article as no new data were created or analyzed in this study.

## Appendix A

*The List of articles that made it through the selection process.*

1. Diamond, L. M., Fagundes, C. P., & Butterworth, M. R. (2012). Attachment style, vagal tone, and empathy during mother–adolescent interactions. Journal of Research on Adolescence, 22(1), 165–184. Retrieved from https://doi.org/10.1111/j.1532-7795.2011.00762.x
2. Kim, S., & Kochanska, G. (2017). Relational antecedents and social implications of the emotion of empathy: evidence from three studies. Emotion, 17(6), 981–992, Retrieved from http://dx.doi.org/10.1037/emo0000297
3. Laible, D. (2007). Attachment with parents and peers in late adolescence: Links with emotional competence and social behavior. *Personality and Individual Differences, 43,* 1185–1197. Retrieved from http://dx.doi.org/10.1016/j.paid.2007.03.010
4. Muris, P., Meesters, C., Cima, M., Verhagen, M., Brochard, N., Sanders, A., Kempener, C. Beurskens, J., & Meesters, V. (2014). Bound to feel bad about oneself: relations between attachment and the self-conscious emotions of guilt and shame in children and adolescents. Journal of Child and Family Studies, 23, 1278–1288. Retrieved from https://doi.org/10.1007/s10826-013-9817-z
5. Murphy, P., Laible, D.J., Augustine, M., & Robeson, L. (2015). Attachment’s links with adolescents’ social emotions: The roles of negative emotionality and emotion regulation. The Journal of Genetic Psychology, 176(5), 315–329. ISSN: 0022-1325 print / 1940-0896 online. Retrieved from: https://doi.org/10.1080/00221325.2015.1072082
6. Paez, A., & Rovella, A. (2019). Vínculo de apego, estilos parentales y empatía en adolescentes. Interdisciplinaria, 36(2), 23-38. Retrieved from: https://doi.org/10.16888/interd.2019.36.2.2
7. Panfile, T., & Laible, D. (2012). Attachment security and child’s empathy: the mediating role of emotion regulation. Merrill-Palmer Quarterly, 58(1), 1-21. Retrieved 16 June 2021, from http://www.jstor.org/stable/23098060
8. Ştefan, C.A, & Avram, J. (2018). The multifaceted role of attachment during preschool: moderator of its indirect effect on empathy through emotion regulation, Early Child Development and Care, 188(1), 62-76. Retrieved from https://doi.org/10.1080/03004430.2016.1246447
9. Stern, J., Costello, M., Kansky, J., Fowler, C., Loeb, E.M, & Allen, J.P. (2021). Here for You: Attachment and the growth of empathic support for friends in adolescence. Child Development, 0(0), 1-16. ISSN: 0009-3920 Online ISSN: 1467-8624. Retrieved from: https://doi.org/10.1111/cdev.13630
10. van der Mark, I., van IJzendoorn, M., & Bakermans-Kranenburg, M.J. (2002). Development of empathy in girls during the second year of life: associations with parenting, attachment, and temperament. Retrieved from https://doi.org/10.1111/1467-9507.00210
